# Assisted Reproductive Technology and Cardiovascular Outcomes in Women: A Systematic Review and Meta-Analysis

**DOI:** 10.3390/jcm15082844

**Published:** 2026-04-09

**Authors:** Shu Qin Wei, Wenwan Li, Nathalie Auger, Brian J. Potter, Gilles Paradis, Jessica Healy-Profitós, Seang-Lin Tan

**Affiliations:** 1 OriginElle Fertility Clinic & Women’s Health Centre, 2110 Dearie Boulevard, Montreal, QC H4A3J3, Canada; swei@originelle.com; 2 Institut National de Santé Publique du Québec, Montreal, QC H2P1E2, Canada; nathalie.auger@inspq.qc.ca (N.A.); gilles.paradis@mcgill.ca (G.P.); jessica.profitos@inspq.qc.ca (J.H.-P.); 3 Department of Epidemiology, Biostatistics and Occupational Health, McGill University, Montreal, QC H3A1Y7, Canada; wenwan.li@mail.mcgill.ca; 4 University of Montreal Hospital Research Centre, Montreal, QC H2X0A9, Canada; brian.potter@umontreal.ca; 5 Department of Medicine, University of Montreal, Montreal, QC H3T1J4, Canada; 6 Department of Obstetrics and Gynecology, McGill University, Montreal, QC H4A0B1, Canada

**Keywords:** assisted reproductive techniques, cardiovascular diseases, fertilization in vitro, infertility, intracytoplasmic sperm injections

## Abstract

**Background:** Assisted reproductive technology has been linked to an increased risk of pregnancy-related cardiovascular complications, but the long-term cardiovascular outcome is poorly understood. This study aimed to assess whether women who use ART have an elevated long-term risk of adverse cardiovascular outcomes. **Methods:** We conducted a systematic review and meta-analysis to examine the association between ART and long-term cardiovascular outcomes after pregnancy. We systematically searched MEDLINE, Embase, and the Cochrane Library for studies published by January 2026. We evaluated the methodological quality of included studies using the Newcastle-Ottawa Scale. We used random effects models to calculate pooled adjusted risk ratios (aRR) with 95% confidence intervals (CI) for the association of ART with cardiovascular outcomes. **Results:** We included thirteen studies comprising 553,331 patients who used ART and 37,826,591 patients who conceived spontaneously. All women achieved a live birth. Mean duration of follow-up after delivery was 8.4 ± 8.3 years. In models adjusted for age, parity, and comorbidity, ART was associated with a small increase in the risk of cardiovascular disease compared with spontaneous conception (aRR 1.18, 95% CI 1.03–1.35), but the association was attenuated when studies that had only 42 days of follow-up were excluded (aRR 1.13, 95% CI 0.99–1.29). ART was not associated with cardiac complications (aRR 0.94, 95% CI 0.82–1.08), stroke (aRR 1.20; 95% CI 0.93–1.55), hypertension (aRR 1.02; 95% CI 0.72–1.44), or venous thrombosis (aRR 1.27, 95% CI 0.97–1.67). **Conclusions:** Our findings suggest that women who achieve a live birth following ART do not appear to have an increased long-term risk of adverse cardiovascular outcomes. These results provide reassuring evidence for patient counseling regarding the long-term cardiovascular safety of ART among women with successful pregnancies. Further research that includes women who do not achieve a live birth is warranted to more fully characterize the potential long-term cardiovascular effects of ART across the entire spectrum of treatment outcomes.

## 1. Introduction

Use of assisted reproductive technology (ART) has increased substantially in the past decade, but the long-term impact on the cardiovascular system is poorly understood. A number of studies have examined the risk of cancer following ART [1,2,3], but the association with cardiovascular disease has received less attention. Cardiovascular disease is a leading cause of morbidity and mortality in women [4], and ART has potential to contribute to cardiovascular risk [5]. ART requires ovarian stimulation procedures that can result in estradiol surges or ovarian hyperstimulation syndrome, complications associated with vascular injury and persistent endothelial dysfunction [5,6]. Ovarian hyperstimulation may affect the renin–angiotensin–aldosterone system and blood pressure regulation [6,7]. Additionally, medications used for ovarian stimulation may elevate estradiol levels and lead to poor distensibility or carotid artery stiffness [8].

Observational studies of the association between ART and cardiovascular disease are inconsistent [7,9]. An Italian study found that women who used ART procedures developed high systolic and diastolic blood pressure more frequently than women who conceived spontaneously [9]. However, an analysis of 116,430 patients from the U.S. found that ART was not associated with the risk of hypertension [7]. The association between ART and outcomes such as stroke and heart failure is also unclear [10]. Previous literature reviews have found that fertility therapy of any type does not increase the long-term risk of cardiovascular disease, but did not focus on ART specifically [10,11]. The meta-analyses in these reports do not specifically isolate the effects of ART [10,11]. Our objective was to systematically review the literature and evaluate the long-term risk of developing cardiovascular disease after delivery of a pregnancy conceived through ART.

## 2. Materials and Methods

This systematic review followed Preferred Reporting Items for Systematic Reviews and Meta-Analyses (PRISMA) guidelines [12]. The completed PRISMA checklist is provided in Table S1. The systematic review was registered in PROSPERO (registration no. CRD42021256461).

### 2.1. Search Strategy

We systematically searched MEDLINE, Embase, and the Cochrane Library for English language studies of the association between ART and cardiovascular outcomes that were published by January 2026. The search terms included the following keywords and medical subject headings: “assisted conception”, “embryo transfer”, “fertilization in vitro”, “reproductive techniques, assisted”, “sperm injections, intracytoplasmic”, “cardiovascular disease”, “heart diseases”, “hypertension”, and “stroke” (Appendix A). To maximize the number of publications identified, we additionally screened the references of published articles for relevant literature.

### 2.2. Study Selection

Two reviewers independently screened titles and abstracts to identify pertinent articles, with any discrepancy resolved by discussion. The reviewers selected full-length articles that met the following inclusion criteria: (1) design was a case–control, cross-sectional, or cohort study; (2) exposure was ART following the definition of the International Committee for Monitoring Assisted Reproductive Technologies [13]; (3) outcomes were cardiovascular diseases; (4) results contained relevant data to obtain the effect size, such as risk ratios or the number of participants; and (5) follow-up began after delivery. We excluded studies that (1) were animal or in vitro analyses; (2) were reviews, case reports, or case series; or (3) lacked a comparison group of patients unexposed to ART.

### 2.3. Exposure

The primary exposure was ART, defined according to the International Committee for Monitoring Assisted Reproductive Technologies glossary [13]. ART included in vitro fertilization, intracytoplasmic sperm injection, embryo biopsy, preimplantation genetic testing, assisted hatching, gamete intrafallopian transfer, zygote intrafallopian transfer, embryo transfer, gamete and embryo cryopreservation, and semen, oocyte or embryo donation.

### 2.4. Outcome

The primary outcome was new onset cardiovascular disease in the long-term period after delivery. Secondary outcomes included cardiac complications (cardiomyopathy, heart failure, myocardial infarction, angina pectoris, inflammatory heart disease, conduction disorders, or other heart disease), stroke (defined as hemorrhagic or ischemic stroke), hypertension, and venous thrombosis.

### 2.5. Assessment of Study Quality

Two reviewers independently assessed the quality of studies using the Newcastle-Ottawa Scale [14]. We scored the overall quality of case–control and cohort studies from 0 to 9. We evaluated case–control studies for representativity of the population, comparability of groups, and ascertainment of exposure [14]. We evaluated cohort studies for representativity of the population, comparability of cohorts, and assessment of outcome [14]. We classified each study as having low (7–9 points), moderate (4–6 points), or high risk of bias (less than 4 points), and assessed publication bias using funnel plots.

### 2.6. Data Extraction and Analysis

Two reviewers independently extracted raw data and summary statistics from each study. We extracted the first author’s last name, publication year, country of origin, study design, follow-up duration, total sample size, exposure, outcomes, and covariates used for adjustment. When relevant data for calculating effect size were unavailable, we excluded the study from meta-analysis.

We estimated the association between ART and risk of cardiovascular disease using random effects models. We performed the analysis for cardiovascular disease of any type and for each outcome separately. We also assessed whether associations varied with length of follow-up after pregnancy, by restricting the analysis to studies in which patients were followed more than 42 days after pregnancy. The purpose was to rule out a contribution of severe maternal morbidity, which by definition includes cardiovascular events up to 42 days after delivery.

We calculated pooled unadjusted and adjusted risk ratios (RR) with 95% confidence intervals (CI) using the inverse variance method. Covariates used for adjustment varied from study to study, but generally included one or more of the following characteristics: maternal age, parity, obesity, diabetes, and chronic hypertension. We used forest plots to graphically present study-specific effect estimates with 95% CIs and displayed pooled RRs as diamonds. We annotated the plots to include study identifiers (first author and year), numbers of events and total participants in the exposed and unexposed groups, study weights, and the estimates. We assessed the heterogeneity of studies with the I^2^ statistic. We considered heterogeneity substantial when the I^2^ was ≥50% following recommendations of the Cochrane Collaboration [15]. We performed data analyses using Review Manager version 5.4 software.

## 3. Results

### 3.1. Search Results

**Figure 1:**
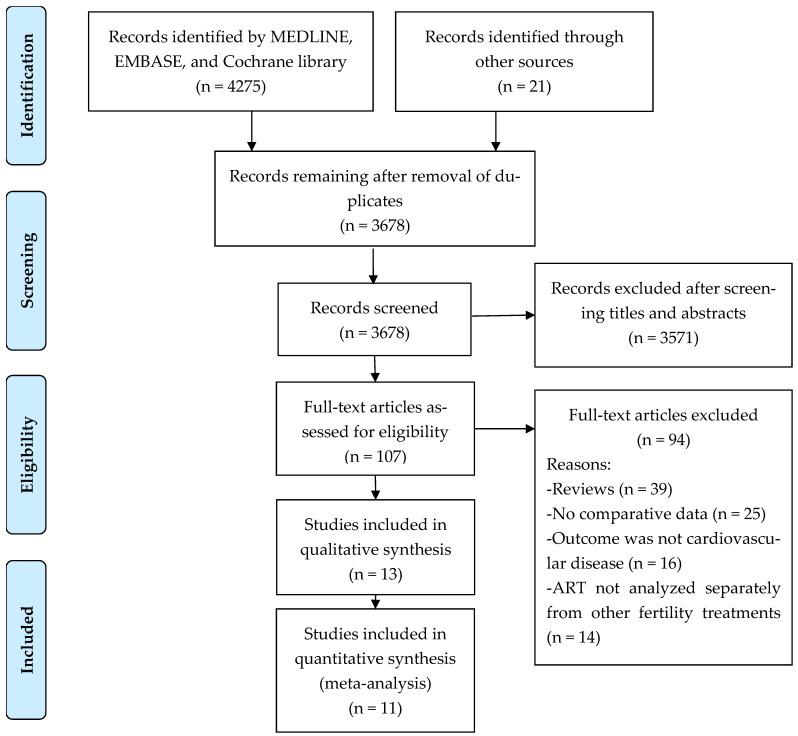
PRISMA flow diagram.

We identified 4275 articles pertaining to cardiovascular complications after use of ART (Figure 1). After excluding 618 duplicate studies, we screened the titles and abstracts of the remaining studies and selected 107 studies for full text review. A total of 13 observational studies involving 553,331 patients who used ART and 37,826,591 who conceived spontaneously met the inclusion criteria for systematic review [7,9,16,17,18,19,20,21,22,23,24,25,26]. Eleven studies were eligible for meta-analysis [7,16,17,19,20,21,22,23,24,25,26]. One of the two ineligible studies did not provide risk estimates [9] and the other [18] used the same population as another study with longer follow-up [17].

### 3.2. Study Characteristics

Studies included in the review involved patients from Canada [20], Denmark [17,18,22], Finland [17,18], Israel [21], Italy [9], Norway [17,18], Sweden [17,18,23,24,25,26], and the United States [7,16,19] (Table 1). One study used a case–control design [9], while the remaining twelve had cohort designs [7,16,17,18,19,20,21,22,23,24,25,26]. One study relied on self-reported data [7], while twelve studies used administrative data from patient registries [9,16,17,18,19,20,21,22,23,24,25,26]. Duration of follow-up for cardiovascular outcomes extended up to 25 years after delivery (mean 8.4 ± 8.3 years). Two studies had a follow-up duration of 42 days postpartum [24,25].

### 3.3. Risk of Bias

Assessment of study quality indicated that risk of bias was low overall, with a mean score of 6.9 (sd 1.9) on the Newcastle-Ottawa Scale (Table S2). Nine studies had a low risk of bias [17,18,19,20,22,23,24,25,26], three had a moderate risk [7,16,21], and one study had a high risk of bias [9]. Funnel plots showed no asymmetry or evidence of publication bias (Figure S1).

### 3.4. Association of ART with Cardiovascular Outcomes

#### 3.4.1. Any Cardiovascular Disease

**Figure 2:**
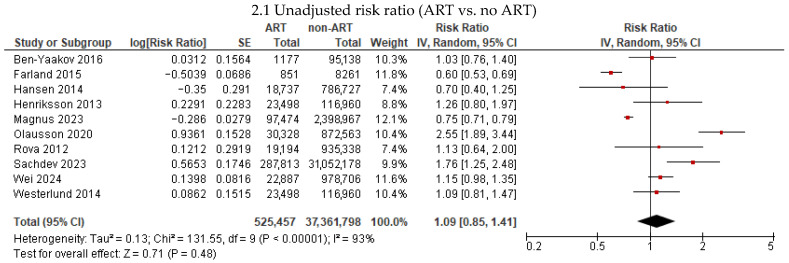

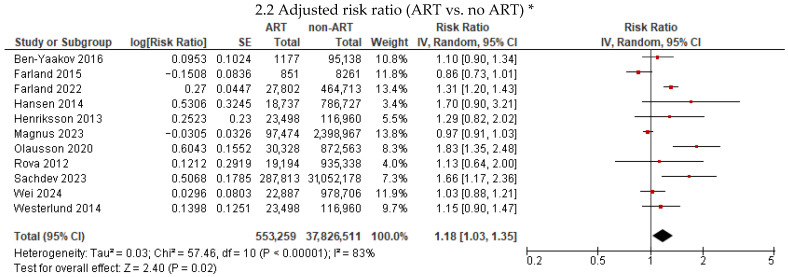
Association between assisted reproductive technology (ART) and any cardiovascular disease. * Adjusted for confounders such as age, parity, obesity, diabetes, and chronic hypertension.

Eleven studies [7,16,17,19,20,21,22,23,24,25,26] reported the association between ART and any cardiovascular disease among 553,259 women who used ART and 37,826,511 women who conceived spontaneously (Figure 2). Compared with spontaneous conception, ART was not associated with an increased risk of any cardiovascular outcome in unadjusted models (RR 1.09, 95% CI 0.85–1.41; I^2^ = 93%; 10 studies). A weak association appeared to be present in models adjusted for age, parity, obesity, diabetes, and chronic hypertension (RR 1.18, 95% CI 1.03–1.35; I^2^ = 83%; 11 studies). However, after excluding 2 studies with only 42 days of follow-up that likely captured peripartum events [24,25], ART was not associated with an increased risk of any cardiovascular outcome in unadjusted (RR 0.97, 95% CI 0.78–1.21; I^2^ = 90%; 8 studies) or adjusted models (RR 1.13, 95% CI 0.99–1.29; I^2^ = 83%; 9 studies) (Figure S2).

#### 3.4.2. Cardiac Complications

**Figure 3:**
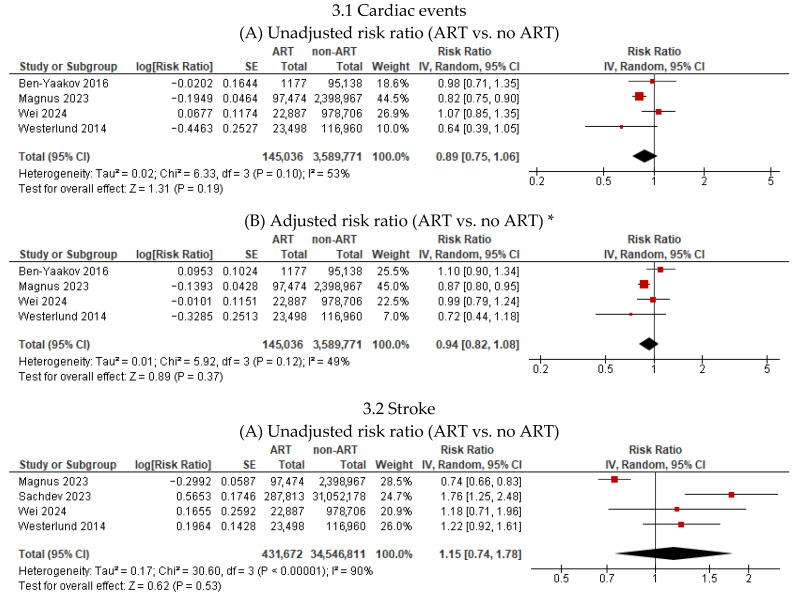

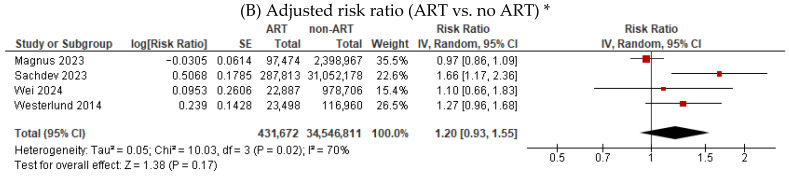
Association of assisted reproductive technology (ART) with cardiac events and stroke. * Adjusted for confounders such as age, parity, obesity, diabetes, and chronic hypertension.

Four studies [17,20,21,26] reported the association between ART and cardiac events among 145,036 women who used ART and 3,589,771 women who conceived spontaneously during a maximum follow-up period of 25 years (Figure 3). Compared with spontaneous conception, ART was not associated with an increased risk of cardiac events in unadjusted (RR 0.89, 95% CI 0.75–1.06; I^2^ = 53%; 4 studies) or adjusted models (RR 0.94, 95% CI 0.82–1.08; I^2^ = 49%; 4 studies).

#### 3.4.3. Stroke

Four studies [17,19,20,26] reported the association between ART and stroke among 431,672 women who used ART and 34,546,811 women who conceived spontaneously during a maximum follow-up period of 18 years (Figure 3). Compared with spontaneous conception, ART was not associated with the risk of stroke in unadjusted (RR 1.15, 95% CI 0.74–1.78; I^2^ = 90%; 4 studies) or adjusted models (RR 1.20, 95% CI 0.93–1.55; I^2^ = 70%; 4 studies).

#### 3.4.4. Hypertension

**Figure 4:**
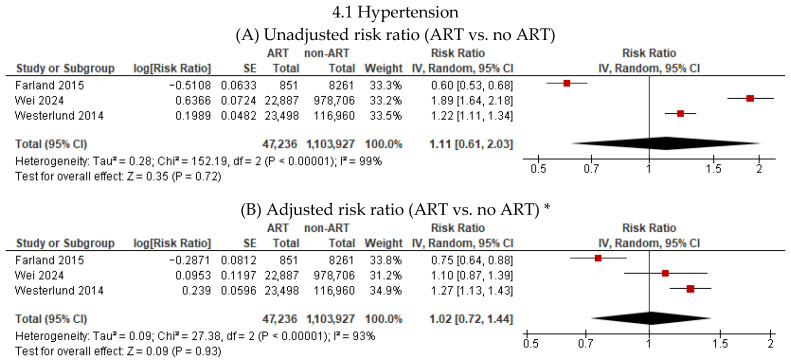

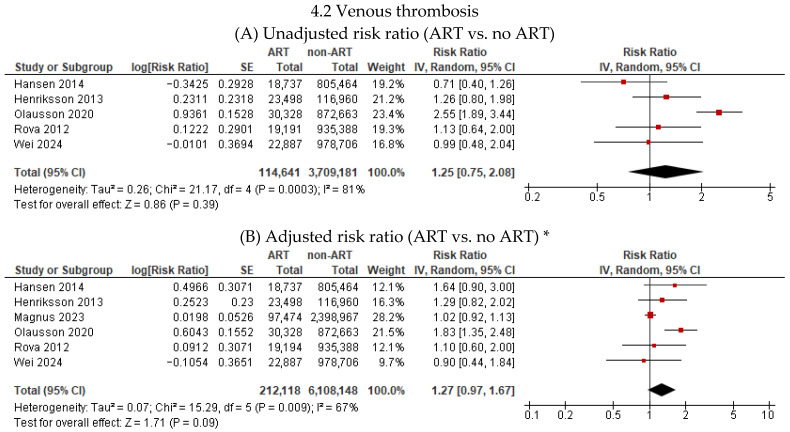
Association of assisted reproductive technology (ART) with hypertension and venous thrombosis. * Adjusted for confounders such as age, parity, obesity, diabetes, and chronic hypertension.

Three studies [7,20,26] reported the association between ART and hypertension among 47,236 women who used ART and 1,103,927 women who conceived spontaneously during a maximum follow-up period of 18 years (Figure 4). Compared with spontaneous conception, ART was not associated with an increased risk of hypertension in unadjusted (RR 1.11, 95% CI 0.61–2.03; I^2^ = 99%; 3 studies) or adjusted models (RR 1.02, 95% CI 0.72–1.44; I^2^ = 93%; 3 studies).

#### 3.4.5. Venous Thrombosis

Six studies [17,20,22,23,24,25] reported the association between ART and venous thrombosis among 212,118 women who used ART and 6,108,148 women who conceived spontaneously during a maximum follow-up period of 19 years (Figure 4). Compared with spontaneous conception, ART was not associated with an increased risk of venous thrombosis in unadjusted (RR 1.25, 95% CI 0.75–2.08; I^2^ = 81%; 5 studies) or adjusted models (RR 1.27, 95% CI 0.97–1.67; I^2^ = 67%; 6 studies).

## 4. Discussion

### 4.1. Main Findings

In this systematic review and meta-analysis of 13 studies comprising 553,331 patients who used ART and 37,826,591 who conceived spontaneously, we found no evidence that ART was associated with the long-term risk of cardiovascular disease later in life compared with spontaneous conception. ART was not associated with cardiac complications, stroke, hypertension, or venous thrombosis up to 25 years after delivery. While studies with longer follow-up may be warranted to confirm these findings, the current evidence suggests that women who deliver an infant conceived through ART do not have an elevated risk of cardiovascular disease in the long term.

### 4.2. Interpretation

Past literature reviews have not examined the association of assisted reproductive technology with later onset of cardiovascular disease directly. Rather, the focus has been fertility treatment in general [10,11]. In these reviews, ART was combined with fertility treatments such as ovarian stimulation drugs, ovulation induction drugs, and intrauterine insemination. The findings suggested that fertility therapy was not associated with an increased risk of cardiovascular disease, but the combination of ART with non-ART procedures prevented the researchers from determining the cardiovascular safety of ART specifically. This issue is problematic as protocols that do not require embryo manipulation are less invasive and unlikely to have cardiovascular sequelae. Artificial insemination generally involves no or only mild ovary stimulation. As less invasive procedures have potential to mask the effect of ART on the cardiovascular system, it is difficult to rule out an association. The present systematic review focuses exclusively on ART and provides more conclusive evidence that ART is not linked with an increased risk of cardiovascular disease in the long term.

Concern about the long-term outcomes of ART may stem from data suggesting that fertility procedures have a cardiovascular impact during pregnancy [7,27]. ART frequently requires repeated cycles of ovarian stimulation with clomiphene or gonadotropins, which studies have linked with hypercoagulability, activation of the renin-angiotensin system, and endothelial dysfunction [7,27]. These sequelae were thought to be the reason why patients who use ART have an elevated risk cardiovascular morbidity during pregnancy [7,27]. A cohort study of 2.3 million pregnant women from Nordic countries found that patients who use frozen embryo transfer are 70% more likely to develop hypertensive disorders of pregnancy [28]. Studies have linked in vitro fertilization with venous thromboembolism among 140,458 pregnant women from Sweden in the first trimester [23], and with severe maternal cardiovascular morbidity among 2.5 million pregnant women from Canada [29].

Other factors have potential to explain why ART might be associated with cardiovascular complications during pregnancy [30,31]. During frozen embryo transfer cycles undertaken with hormone replacement therapy, patients do not develop a corpus luteum, the temporary structure formed by the ovarian follicle following ovulation [30,31]. The corpus luteum produces reproductive hormones meant to prepare the endometrium for implantation and the early stages of pregnancy [30]. The corpus luteum also secretes relaxin, a vasodilator that lowers blood pressure and helps the maternal cardiovascular system adapt to pregnancy [30]. A study of 65 women who conceived with in vitro fertilization found that patients who did not have a corpus luteum had significantly reduced cardiac output, left atrial dimension, and arterial compliance in early pregnancy compared with women who had a corpus luteum [30]. A study of 71 patients found that women who underwent frozen embryo transfer without a corpus luteum had blunted carotid-femoral pulse flow and higher rates of preeclampsia compared with patients who conceived using other forms of ART [31].

Although there are biologically plausible pathways linking ART with cardiovascular complications during gestation, a long-term cardiovascular effect appears less likely. In our review, there was no association between ART and cardiovascular morbidity up to 25 years after pregnancy. The absence of an association over the long term is nevertheless surprising, as patients who use ART commonly have cardiovascular risk factors. Advanced maternal age and comorbidities such as obesity, diabetes, polycystic ovary syndrome, or other endocrine disorders known to increase cardiovascular risk are common in patients receiving ART [10]. Many of the studies included in our review were unable to account for all these factors. While some studies adjusted for age and certain comorbidities [16,17,20,21], others accounted for a more limited number of risk factors [7,19,23,24,26]. One study did not adjust for any confounders [9] and two did not clearly describe the factors they adjusted for [22,25]. Some degree of residual confounding is therefore expected to have led to an association between ART and subsequent cardiovascular morbidity. However, there was limited evidence of a link in most studies.

The absence of an expected association between ART and cardiovascular disease could be due to mitigating factors that attenuate risks. Women who use ART often have higher socioeconomic status and healthier lifestyles, both of which are linked to a lower risk of cardiovascular disease [32]. In addition, the reason for pursuing ART may relate to the partner and have no direct cardiovascular implication for a patient. For instance, male factor infertility has no direct relationship with a woman’s cardiovascular risk, but accounts for a significant proportion of ART procedures [33]. These factors could potentially mask or attenuate any overall effect of ART on long-term cardiovascular health, if one exists. For this reason, results of the studies included in this review should be met with caution. Continued vigilance and research may be needed to ensure there is indeed no impact of ART on subsequent cardiovascular risk.

### 4.3. Strengths and Limitations

The studies included in our review focused exclusively on ART procedures that led to live births. Patients who do not achieve pregnancy or have early losses are not represented in these studies. Only about 30% of patients who undergo ART achieve a live birth [34]. The remaining 70% who do not have successful pregnancies are more likely to have comorbidities such as obesity or polycystic ovary syndrome that could potentiate any cardiovascular impact of ART procedures [34,35,36]. In a study of ovulation induction among 28,442 patients who received gonadotropins or gonadotropin-releasing hormones, patients who did not achieve a live birth were 21% more likely to develop cardiovascular disease up to 22 years later compared with patients who successfully gave birth [34]. The excess risk was mainly driven by heart failure and cerebrovascular disease [34]. While the study compared women with unsuccessful ART to those who achieved a live birth following ART, the findings nevertheless support the possibility that patients who do not achieve pregnancy or a live birth after using ART may be at risk of cardiovascular sequelae [34]. More research is needed to determine if women whose ART attempts do not result in live births are at risk of long-term cardiovascular morbidity.

This systematic review has additional limitations. Several of the included studies relied on administrative data, which may be subject to nondifferential misclassification due to random errors in variable reporting. Owing to lack of data, we could not assess the underlying causes of infertility, specifics of ART cycles, or the types of ART, all of which could potentially affect cardiovascular outcomes differently. The observational nature of the data also means that we cannot rule out residual confounding. The relatively high heterogeneity observed in some analyses possibly reflects differences in study settings, population characteristics, handling of infertility-related factors, and the extent of adjustment for confounders. The findings are representative only of patients who achieved a live birth, not patients who had early losses or never conceived. Lastly, the review was restricted to studies published in English.

## 5. Conclusions

In this systematic review of 13 studies comprising 553,331 patients who used ART and 37,826,591 patients who conceived spontaneously, ART was not associated with an increased risk of adverse cardiovascular outcomes up to 25 years after pregnancy. The findings suggest that ART is unlikely to have a long-term cardiovascular impact among women who successfully give birth and may help guide patient counseling by providing reassurance regarding future cardiovascular health. Future studies of patients who do not achieve a live birth may be merited to fully understand the potential long-term impact of ART on cardiovascular outcomes.

## Figures and Tables

**Table 1:** Characteristics of included studies.

Study	Country	Study Design	Study Period	Follow-Up Duration	No. Patients	Population	Comparison	Outcome	Confounders
Ben-Yaakov 2016 [21]	Israel	Retrospective cohort	1998–2012	Up to 25 (Mean 11.7) years	99,291	Women who received IVF	Women who did not use fertility treatment	Cardiovascular hospitalization	Age, parity, preeclampsia, diabetes, obesity
Farland 2015 [7]	United States	Prospective cohort	1993–2011	Up to 18 years	116,430	Women who received IVF	Women who did not use fertility treatment	Hypertension	Age, body mass index, race, smoking, income, parity, history of sexual abuse
Farland 2022 [16]	United States	Retrospective cohort	2004–2017	Up to 8 years	492,515	Women who received ART	Women who did not use fertility treatment	Cardiovascular disease	Age, parity, year of delivery, plurality, chronic hypertension before pregnancy, chronic diabetes before pregnancy
Hansen 2014 [22]	Denmark	Retrospective cohort	1995–2005	12 weeks postpartum	18,787	Women who received IVF	Women without IVF treatment	Venous thrombosis	Confounders for the adjusted model were not described.
Henriksson 2013 [23]	Sweden	Retrospective cohort	1990–2008	1 year	140,458	Women who received IVF	Women with natural conception	Pulmonary embolism, venous thromboembolism	Age, calendar year of delivery, body mass index, parity, smoking, country of birth, marital status, education
Magnus 2023 [17]	Denmark Finland Norway Sweden	Retrospective cohort	1984–2015	Median 11 (IQR, 5–18) years	2,496,441	Women who received ART	Women without fertility treatment	Cardiovascular disease	Age, calendar year at start of follow-up, parity, polycystic ovary syndrome, diabetes, chronic hypertension, country
Magnus 2024 [18]	Denmark Finland Norway Sweden	Retrospective cohort	1984–2015	During the first year after delivery	2,496,441	Women who received ART	Women without fertility treatment	Stroke	Age, calendar year at start of follow-up, parity, polycystic ovary syndrome, diabetes, chronic hypertension, country
Olausson 2020 [24]	Sweden	Retrospective cohort	1992–2012	42 days postpartum	902,891	Women who received IVF with fresh or frozen-thawed embryo transfer	Women with natural conception	Pulmonary embolism, venous thromboembolism	Age, calendar year of delivery, body mass index, education, smoking, country of birth
Rosato 2016 [9]	Italy	Case–control	2010–2014	3 years	152	Women ≥ 43 years who received ART	Women ≥ 43 years who conceived spontaneously	Systolic blood pressure, diastolic blood pressure, hypertension, diabetes	None
Rova 2012 [25]	Sweden	Retrospective cohort	1999–2008	42 days postpartum	964,532	Women who received IVF	Women without ART treatment	Venous thromboembolism	Confounders for the adjusted model were not described.
Sachdev 2023 [19]	United States	Retrospective cohort	2010–2018	1 year after delivery	31,339,991	Women who received ART	Women without ART treatment	Stroke hospitalization	Maternal age, multiple birth, hospital type, hospital teaching status, income quartile, insurance, year of hospital discharge
Wei 2024 [20]	Canada	Retrospective cohort	2008–2019	Up to 11 years	1,001,593	Women who received ART	Women without ART treatment	Cardiovascular hospitalization	Maternal age, parity, maternal comorbidity, socioeconomic disadvantage, home language, parental place of birth, year of birth
Westerlund 2014 [26]	Sweden	Retrospective cohort	1990–2008	Mean 8.6 years	140,458	Women who received IVF	Women with natural conception	Hypertension, stroke, coronary heart disease	Body mass index, smoking, country of birth, education

Abbreviations: ART = assisted reproductive technology; IVF = in vitro fertilization.

## Data Availability

The data underlying this article are available in the article.

## Supplementary Materials

The following supporting information can be downloaded at: https://www.mdpi.com/article/10.3390/jcm15082844/s1, Figure S1: Funnel plots for publication bias; Figure S2. Association between assisted reproductive technology (ART) and any cardiovascular disease, excluding Olausson 2020 [24] and Rova 2012 [25] that had follow-up periods of up to 42 days after delivery; Table S1: PRISMA 2020 Checklist; Table S2: Newcastle-Ottawa Scale for risk of bias.

## Appendix A

### Search Strategy

Medline

1 exp reproductive techniques, assisted
2 assisted reproducti* technique*.mp.
3 assisted reproducti* technolog*.mp.
4 donor conception.mp.
5 embryo transfer*.mp.
6 fertility preservation.mp.
7 fertilization in vitro.mp.
8 gamete intrafallopian transfer*.mp.
9 in vitro oocyte maturation technique*.mp.
10 oocyte donation.mp.
11 oocyte retrieval.mp.
12 ovulation induction.mp.
13 posthumous conception.mp.
14 sperm retrieval.mp.
15 zygote intrafallopian transfer.mp.
16 1 or 2 or 3 or 4 or 5 or 6 or 7 or 8 or 9 or 10 or 11 or 12 or 13 or 14 or 15
17 exp heart diseases
18 hypertension*.mp.
19 stroke.mp.
20 exp vascular diseases
21 17 or 18 or 19 or 20
22 16 and 21

Embase

1 exp infertility therapy
2 assisted reproducti* technique*.mp.
3 assisted reproducti* technolog*.mp.
4 donor conception.mp.
5 embryo transfer*.mp.
6 fertility preservation.mp.
7 infertility therapy.mp.
8 gamete intrafallopian transfer.mp.
9 in vitro fertilization.mp.
10 oocyte donation.mp.
11 zygote intrafallopian transfer.mp.
12 ovulation induction.mp.
13 cardiovascular disease
14 heart disease
15 stroke.mp.
16 1 or 2 or 3 or 4 or 5 or 6 or 7 or 8 or 9 or 10 or 11 or 12
17 13 or 14 or 15
18 16 and 17

Cochrane library

1 MeSH descriptor: [reproductive techniques, assisted] explode all trees
2 ivf
3 in vitro fertilization
4 icsi
5 intracytoplasmic sperm injection
6 embryo transfer
7 gamete intrafallopian transfer
8 zygote intrafallopian transfer
9 MeSH descriptor: [cardiovascular diseases] explode all trees
10 1 or 2 or 3 or 4 or 5 or 6 or 7 or 8 or 9
11 9 and10
