# Supplementary material for: Assisted Reproductive Technology and Cardiovascular Outcomes in Women: A Systematic Review and Meta-Analysis
Source: J Clin Med. 2026 Apr 9;15(8):2844. doi: 10.3390/jcm15082844 (PMC13116687; doi:10.3390/jcm15082844)
Supplement: Supplementary file 1 [file jcm-15-02844-s001.zip › jcm-4226846-supplementary_2026.3.16.pdf]

Table S2. Newcastle-Ottawa Scale for risk of bias.

Study	Selection	Comparability	Exposure / Outcome	Total no. stars
Ben-Yaakov 2016	★★★	★	★★	6
Farland 2015	★★	★	★	4
Farland 2022	★★★	★	★★	6
Hansen 2014	★★★	★★	★★	7
Magnus 2023	★★★	★★	★★★	8
Magnus 2024	★★★	★★	★★★	8
Henriksson 2013	★★★★	★	★★★	8
Olausson 2020	★★★	★★	★★★	8
Rosato 2016	★	★	★	3
Rova 2012	★★★★	★★	★★★	9
Sachdev 2023	★★★★	★★	★★★	9
Wei 2024	★★★★	★★	★★★	9
Westerlund 2014	★★★★	★★	★★★	9

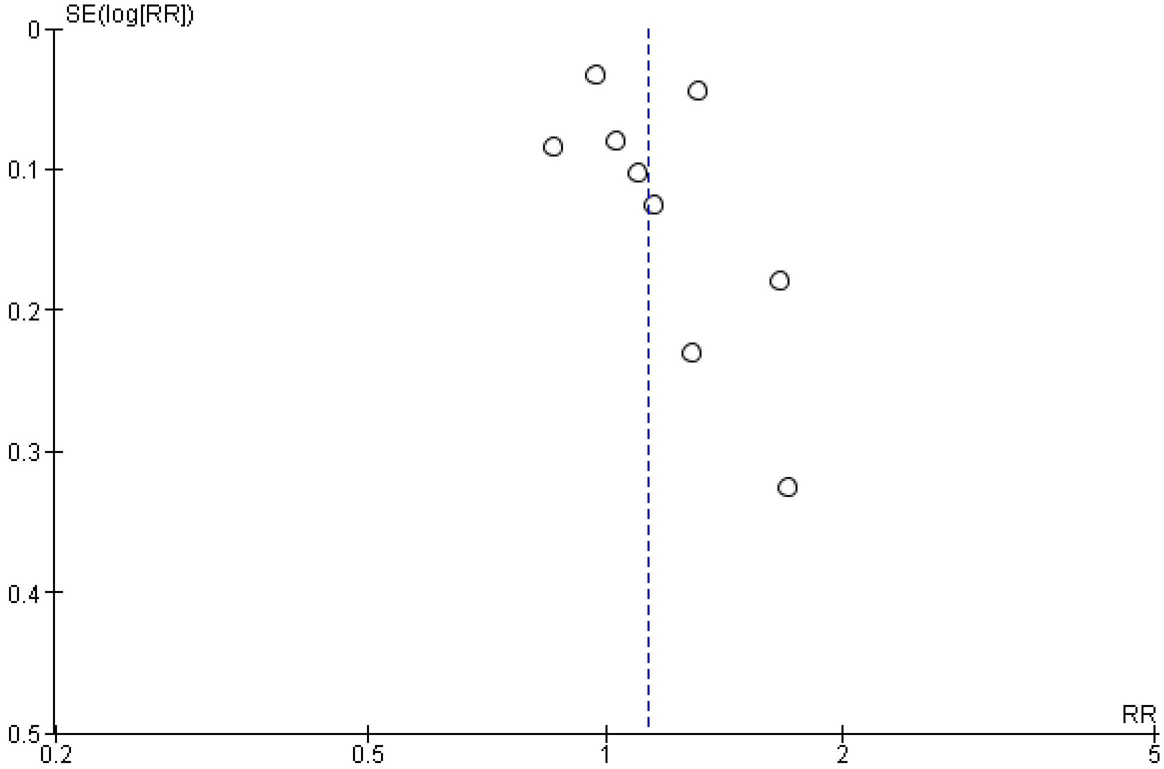

Figure S1. Funnel plots for publication bias.

S2.1 Unadjusted risk ratio (ART vs. no ART)

Study or Subgroup	log[Risk Ratio]	SE	ART Total	non-ART Total	Weight	Risk Ratio IV, Random, 95% CI
Ben-Yaakov 2016	0.0312	0.1564	1177	95138	12.3%	1.03 [0.76, 1.40]
Farland 2015	-0.5039	0.0686	851	8261	15.2%	0.60 [0.53, 0.69]
Hansen 2014	-0.35	0.291	18737	786727	7.8%	0.70 [0.40, 1.25]
Henriksson 2013	0.2291	0.2283	23498	116960	9.7%	1.26 [0.80, 1.97]
Magnus 2023	-0.286	0.0279	97474	2398967	16.0%	0.75 [0.71, 0.79]
Sachdev 2023	0.5653	0.1746	287813	31052178	11.6%	1.76 [1.25, 2.48]
Wei 2024	0.1398	0.0816	22887	978706	14.9%	1.15 [0.98, 1.35]
Westerlund 2014	0.0862	0.1515	23498	116960	12.5%	1.09 [0.81, 1.47]
**Total (95% CI)**			**475935**	**35553897**	**100.0%**	**0.97 [0.78, 1.21]**

Heterogeneity: Tau² = 0.08; Chi² = 72.46, df = 7 (P < 0.00001); I² = 90%
Test for overall effect: Z = 0.23 (P = 0.81)

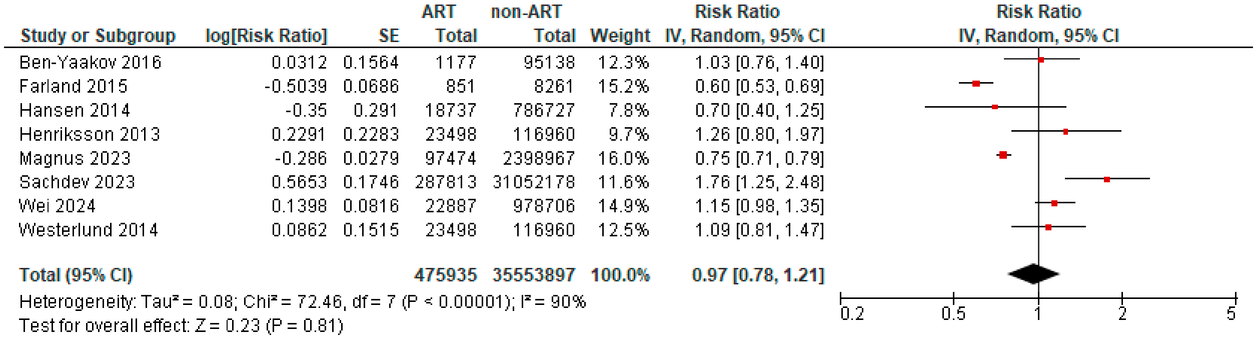

S2.2 Adjusted risk ratio (ART vs. no ART)*

Study or Subgroup	log[Risk Ratio]	SE	ART Total	non-ART Total	Weight	Risk Ratio IV, Random, 95% CI
Ben-Yaakov 2016	0.0953	0.1024	1177	95138	12.3%	1.10 [0.90, 1.34]
Farland 2015	-0.1508	0.0836	851	8261	13.6%	0.86 [0.73, 1.01]
Farland 2022	0.27	0.0447	27802	464713	15.8%	1.31 [1.20, 1.43]
Hansen 2014	0.5306	0.3245	18737	786727	3.6%	1.70 [0.90, 3.21]
Henriksson 2013	0.2523	0.23	23498	116960	5.9%	1.29 [0.82, 2.02]
Magnus 2023	-0.0305	0.0326	97474	2398967	16.3%	0.97 [0.91, 1.03]
Sachdev 2023	0.5068	0.1785	287813	31052178	7.9%	1.66 [1.17, 2.36]
Wei 2024	0.0296	0.0803	22887	978706	13.8%	1.03 [0.88, 1.21]
Westerlund 2014	0.1398	0.1251	23498	116960	10.9%	1.15 [0.90, 1.47]
**Total (95% CI)**			**503737**	**36018610**	**100.0%**	**1.13 [0.99, 1.29]**

Heterogeneity: Tau² = 0.03; Chi² = 45.75, df = 8 (P < 0.00001); I² = 83%
Test for overall effect: Z = 1.77 (P = 0.08)

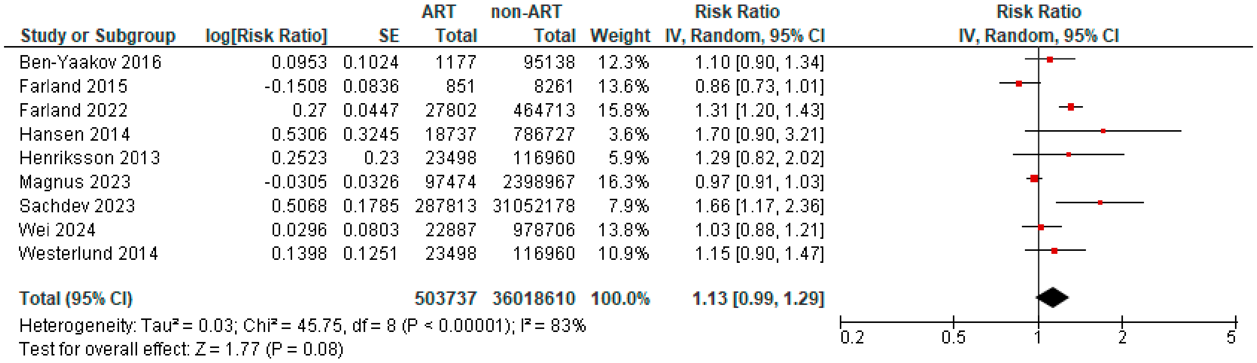

Figure S2. Association between assisted reproductive technology (ART) and any cardiovascular disease, excluding Olausson 2020 [24] and Rova 2012 [25] that had follow-up periods of up to 42 days after delivery. *Adjusted for confounders such as age, parity, obesity, diabetes, and chronic hypertension.
